# Alendronate and omeprazole in combination reduce angiogenic and growth signals from osteoblasts

**DOI:** 10.1016/j.bonr.2021.100750

**Published:** 2021-01-26

**Authors:** Tormod B. Krüger, Bente B. Herlofson, Aina M. Lian, Unni Syversen, Janne E. Reseland

**Affiliations:** a Department of Oral Surgery and Oral Medicine, Faculty of Dentistry, University of Oslo, Norway; b Clinical Oral Research Laboratory, Faculty of Dentistry, University of Oslo, Norway; c Department of Clinical and Molecular Medicine, Faculty of Medicine and Health Sciences, NTNU—Norwegian University of Science and Technology, 7491 Trondheim, Norway; d Department of Endocrinology, Clinic of Medicine, St. Olavs University Hospital, 7491 Trondheim, Norway

**Keywords:** Bisphosphonates, Alendronate, Proton pump inhibitors, Omeprazole, Osteonecrosis, Bone repair

## Abstract

**Objective:**

Due to gastrointestinal side effects of oral bisphosphonates (BPs), proton pump inhibitors (PPIs) are often prescribed. PPIs may enhance the risk of osteonecrosis of the jaw, a rare side effect of BPs. Therefore, the objective of this study was to evaluate the effects of the oral BP alendronate (ALN) and the PPI omeprazole (OME) alone and in combination on primary human osteoblasts and gingival fibroblasts in vitro.

**Methods:**

Human gingival fibroblasts and normal human osteoblasts were incubated with either 5 μM of ALN or 1 μM of OME, or ALN + OME for 1, 3, 7 or 14 days. Effect on viability was evaluated by the lactate dehydrogenase activity in the medium and on proliferation by quantifying 3H-thymidin incorporation. Multianalyte profiling of proteins in cell culture media was performed using the Luminex 200TM system to assess the effect on selected bone markers and cytokines.

**Results:**

The proliferation of osteoblasts and fibroblasts was reduced upon exposure to ALN + OME. ALN induced an early, temporary rise in markers of inflammation, and OME and ALN + OME promoted a transient decline. An initial increase in IL-13 occurred after exposure to all three options, whereas ALN + OME promoted IL-8 release after 7 days. OME and ALN + OME promoted a transient reduction in vascular endothelial growth factor (VEGF) from osteoblasts, whereas ALN and ALN + OME induced a late rise in VEGF from fibroblasts. Osteoprotegerin release was enhanced by ALN and suppressed by OME and ALN + OME.

**Conclusions:**

ALN + OME seemed to exaggerate the negative effects of each drug alone on human osteoblasts and gingival fibroblasts. The anti-proliferative effects, modulation of inflammation and impairment of angiogenesis, may induce unfavorable conditions in periodontal tissue facilitating development of osteonecrosis.

## Highlights

• Alendronate and omeprazole reduce proliferation in osteoblasts and fibroblasts.
• Unchanged viability after exposure to either drug or the combination
• Omeprazole, alone and combined with alendronate, cause impairment of angiogenesis.
• Alendronate promotes an initial, transient increase in pro-inflammatory cytokines.

## 1 Introduction

Adverse effects of bisphosphonates (BPs), like osteonecrosis of the jaw (ONJ), have got increasing attention in the last decade (Yang et al., 2019; Yarom et al., 2019). BP-related osteonecrosis of the jaw (BRONJ) was first described in cancer patients receiving high dose IV BPs, and subsequently also in osteoporosis patients treated with oral or IV BPs, preferentially amino-BPs (Marx, 2003). Alendronate (ALN) given orally is the most widely used BP in treatment of osteoporosis (Pazianas et al., 2007). The incidence of BRONJ among patients with osteoporosis is very low, ranging from 0.02–0.4%, however, an underestimation has been suggested (Galis et al., 2017; Hansen et al., 2013). Medications other than BPs, such as denosumab and antiangiogenic drugs, have also been related to ONJ (Hansen et al., 2013), and the term medication- related ONJ (MRONJ) is applied to cover all drugs (Migliorati et al., 2019).

The pathophysiology of BRONJ is not settled, and it is unknown whether necrosis precedes or follows infection. Several authors have proposed that it may be attributed to over-suppression of osteoclasts by long-term BP therapy, resulting in impairment of osteoblast function and bone renewal (Aghaloo et al., 2015). BPs have been reported to exert an anti-proliferative effect on osteoblasts (Koch et al., 2010), the data are, however, diverging (Krüger et al., 2016). Impairment of angiogenesis is another factor suggested in the pathophysiology. Accordingly, treatment with BPs has been reported to reduce vascular endothelial growth factor (VEGF) (Santini et al., 2002). Moreover, the inflammation induced by BPs is postulated to promote BRONJ (Endo et al., 2017). Healing of oral soft tissue has also been shown to be affected in a negative manner after treatment with BPs. The migration and growth capacity of oral fibroblasts were blocked, as well as a downregulation of type-1 collagen, which is necessary for re-epithelization (Ravosa et al., 2011). Under these circumstances, events such as tooth extractions and dental infections might result in tissue death, vascular loss, and eventually osteonecrosis (Wan et al., 2020).

Comorbidity and certain drugs like glucocorticoids seem to enhance the risk of BRONJ (Khan et al., 2015). H2-blocking agents have also been mentioned in this context (Rejnmark, 2008). In a Danish study including more than 60,000 subjects using ALN, use of proton pump inhibitors (PPIs) was independently associated with surgically treated BRONJ (Eiken et al., 2017). ONJ was also observed in a patient using the PPI esomeprazole without simultaneous BP therapy (Marconcini et al., 2019). Concomitant treatment with BPs and PPIs has also been reported to increase the risk of atypical femur fractures, another rare adverse effect of BPs (Giusti et al., 2010). PPIs are often prescribed to patients treated with oral BPs, as gastrointestinal complaints are the most common adverse effects. Use of PPIs is associated with a modest increase in fracture risk, whereas few studies have shown a reduction in bone mineral density (BMD) (Ozdil et al., 2013).

At a cellular level, esomeprazole, lansoprazole and omeprazole (OME) have been observed to exert inhibitory effects on osteoclasts and osteoblasts at concentrations similar to the plasma levels attained with therapeutic dosages (Costa-Rodrigues et al., 2013). These results suggest that PPIs might have a direct deleterious effect on bone cells, with the possibility of decreased bone turnover. OME has been reported to stimulate osteoblast proliferation, but keeping the level of mineralization unchanged (Salai et al., 2013). There are few, if any, in vitro studies addressing the effect of BPs and PPIs on human gingival fibroblasts, or osteoblasts.

Hence, the aim of this study was to investigate the effect of ALN and OME alone and in combination on human osteoblasts and gingival fibroblasts, addressing cell viability, proliferation and secretion of selected bone markers and cytokines.

## 2 Materials and methods

### 2.1 Study design

Commercially available cells were used in all aspects of this study. Primary human osteoblasts at passage 4 from tibia of a one-day old female donor (Cambrex BioScience, Walkersville, MD, USA) were grown in Lonza Osteoblast Growth Media (OGM) (Cambrex BioScience), containing ascorbic acid, fetal calf serum and gentamicin.

Human gingival fibroblasts, passage 4 (LGC Standards GmbH, Mercatorstr. 51, 46485 Wesel, Germany) isolated from a 28-year-old male donor were cultivated with Lonza Fibroblast Growth Media (FGM) (Cambrex BioScience) containing fetal calf serum and gentamicin. Cells were subcultured at 37 °C in a humidified atmosphere of 5% CO_2_ prior to confluence according to manufacturers' instructions.

Cells were seeded in 12-well plates and incubated with ALN and/or OME (Sigma-Aldrich Biotechnology, Saint Louis, MO, USA) dissolved in OGM/FGM at concentrations of 5 and 1 μM, respectively (*n* = 3). Cells and cell culture media were harvested after 1, 3, 7 or 14 days of incubation, with the last medium change, with or without factors, 24 h prior to harvest. Unexposed cells were used as control at each time point.

### 2.2 Cell viability and proliferation

Cell viability was evaluated by monitoring the activity of lactate dehydrogenase (LDH) in the cell culture medium. The LDH was measured using the microplate-based Cytotoxicity Detection Kit (LDH; Boehringer, Mannheim, Germany). In accordance with the manufacturers' protocol, 50 μl aliquots of cell culture medium were used and the absorbance was read using a microplate reader (Elx800, BioTek, Bad Friedrichshall, Germany) at 450 nm.

The proliferation rate of the cells (approx. 5 × 10^3^ cells/cm^2^) was measured by [3H]-thymidine incorporation into the new strands of DNA during replication. Sub confluent cells were incubated with cell culture medium containing either 5 μM of ALN, 1 μM of OME, or a combination of these drugs. Unexposed cells were used as control at each time point. The cells were pulsed with 1 μCi ^3^H-thymidine/well 12 h prior to harvest. The medium was removed, and the cells were washed twice with phosphate-buffered saline (PBS) and twice with 5% trichloroacetic acid (TCA) to remove unincorporated [3H]-thymidine. The cells were solubilized in 500 μl of 1 M sodium hydroxide (NaOH), and 200 μl of the solubilized cell solution was transferred to 4 ml scintillation fluid (Lumagel; Lumac LSC BV; Packard, Groningen, Netherlands) and counted for 3 min in a liquid scintillation counter (Packard 1900 TR, Packard Instruments, Meriden, CT, USA).

### 2.3 Quantification of specific proteins in cell culture medium

Prior to analysis, the cell culture medium was concentrated five times using Microsep Centrifugal tubes with 3-kDa cut-off (Pall Life Science, Ann Armor, MI, USA).

Multianalyte profiling was performed using the Luminex 200TM system (Luminex Corporation, Austin, TX, USA) and the XY-platform, and acquired fluorescence data were analyzed by the 3.1 x PONENT software (Luminex).

The concentrations of cytokines in cell culture media were determined using the 29-Milliplex Human Cytokine Immunoassay kit (Millipore, Billerica, MA, USA). The cytokines include epidermal growth factor (EGF), eotaxin, granulocyte colony-stimulating factor (G-CSF), granulocyte-macrophage colony-stimulating factor (GM-CSF), interferon alpha-2 (IFN-a2), IFN-g, interleukin-10 (IL-10), IL-12p40, IL-13, IL-15, IL-17, IL-1ra, IL-1a, IL-1b, IL-2, IL-3, IL-4, IL-5, IL-6, IL-7, IL-8, interferon gamma-induced protein 10 (IP-10), monocyte chemoattractant protein-1 (MCP-1), macrophage inflammatory protein-1a (MIP-1a), MIP-1b, tumor necrosis factor-a (TNF-a), TNF-b and vascular endothelial growth factor (VEGF). Further, the level of bone markers IL-6, OPG, osteocalcin (OC), leptin, TNF-a, sclerostin, fibroblast growth factor 23 (FGF-23) were determined using Milliplex Human Bone Panel Immunoassay kit, HBNMAG-51K-7plex (Millipore). All analyses were performed according to the manufacturers' protocols.

### 2.4 Statistical analysis

Statistical evaluation was performed using the software SigmaPlot version 13.0 and 14.0 (Systat Software, San Jose, CA, USA). Statistical significance was assessed by Student's *t*-test and *P*-value set to 0.05, all data groups passed tests for normality and equality.

## 3 Results

### 3.1 Viability and proliferation

ALN, OME and ALN + OME induced no changes in cell viability of either cell type tested after three days of incubation compared to controls (Table 1). Administration of ALN and OME reduced the proliferation of osteoblasts at day 1, followed by an enhancement to control levels at day 3 and a reduction at day 7. ALN + OME, however, caused a time dependent reduction in osteoblast proliferation to 34% (*p* = 0.001) of control at day 7. The proliferation of fibroblasts was reduced to 81% (*p* = 0.041) of control when exposed to ALN + OME (Table 2).

**Table 1:** Lactate dehydrogenase (LDH) activity in cell culture medium presented in % of control after exposure to ALN, OME and ALN + OME.

Osteoblasts						
Day	Alendronate 5 μM	P-value	Omeprazole 1 μM	*P*-value	Alendronate + omeprazole	P-value
1	100.5 ± 2.6	0.872	95.8 ± 2.1	0.140	99.1 ± 0.9	0.492
3	103.4 ± 2.9	0.378	93.3 ± 3.4	0.150	98.1 ± 3.6	0.658


Fibroblasts						
Day	Alendronate 5 μM	P-value	Omeprazole 1 μM	P-value	Alendronate + omeprazole	P-value
1	107.9 ± 2.6	0.070	102.4 ± 2.2	0.442	98.8 ± 1.2	0.609
3	99.4 ± 3.0	0.866	98.9 ± 3.5	0.770	101.1 ± 1.7	0.580

The effect was measured after 1 and 3 days of incubation with 5 μM ALN, 1 μM OME and ALN + OME.

**Table 2:** Effects of ALN, OME and ALN + OME on cell proliferation, measured by [3H]-thymidine incorporation.

Osteoblasts						
Day	Alendronate 5 μM	*P*-value	Omeprazole 1 μM	P-value	Alendronate + omeprazole	P-value
1	55.9 ± 3.1	0.007	51.4 ± 2.3	0.004	65.6 ± 14.6	0.108
3	103.6 ± 22.2	0.881	140.9 ± 30.6	0.259	58.1 ± 5.6	0.006
7	41.2 ± 2.6	0.001	79.3 ± 6.6	0.08	33.8 ± 3.1	0.001


Fibroblasts						
Day	Alendronate 5 μM	P-value	Omeprazole 1 μM	*P*-value	Alendronate + omeprazole	P-value
1	91.2 ± 2.5	0.037	87.3 ± 3.7	0.031	84.6 ± 6.8	0.090
3	92.7 ± 2.9	0.214	96.7 ± 21.9	0.891	84.2 ± 3.1	0.036
7	98.9 ± 6.6	0.896	92.7 ± 4.1	0.301	81.4 ± 4.3	0.041

Subconfluent cells incubated for 1, 3 or 7 days with 5 μM ALN, 1 μM OME and the drugs combined. Data are presented in % relative to control at each time point.

### 3.2 Factors affecting angiogenesis

**Fig. 1:**
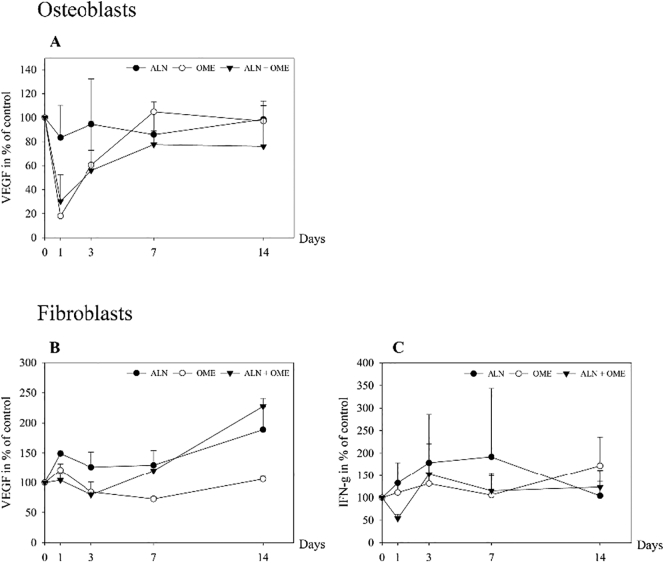
Measured levels of vascular endothelial growth factor (VEGF) (A) in cell culture media from human osteoblasts and VEGF (B) and interferon gamma (INF-g) (C) levels in cell culture media from human gingival fibroblasts. The cells were exposed to 5 μM ALN, 1 μM OME and ALN + OME. Data are presented in % relative to untreated control at each time point.

An immediate decline in secretion of VEGF from osteoblasts to 20% of control was observed when incubated with OME (*p* = 0.011), and with ALN + OME to 30% of control (*p* = 0.030), whereas there was no significant effect of ALN (Fig. 1A). Secretion of VEGF from the fibroblasts was more than doubled after 14 days of administration of ALN + OME (*p* ≤ 0.001), while at the same time point ALN caused increase in secretion to 170% of control (*p* = 0.003). In contrast, OME alone did not result in any significant changes in VEGF secretion (Fig. 1B). ALN + OME induced a transient reduction in secretion of IFN-g from fibroblasts after one day of incubation (*p* = 0.013). A non-significant rise occurred after exposure to ALN for three days and OME for 14 days (Fig. 1C).

### 3.3 Factors affecting osteoclastogenesis

**Fig. 2:**
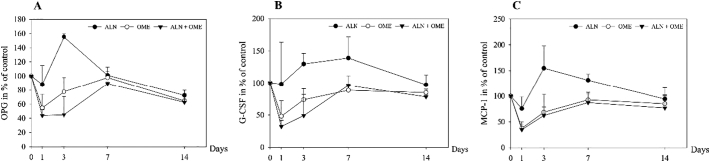
Measured levels of osteoprotegerin (OPG) (A), granulocyte colony-stimulating factor (G-CSF) (B) and monocyte chemoattractant protein (MCP-1) (C) in cell culture media from human osteoblasts. The cells were exposed to 5 μM ALN, 1 μM OME and ALN + OME. Data are presented in % relative to unexposed control at each time point.

After 24 h, the secretion of OPG from osteoblasts incubated with OME was reduced to 55% (*p* = 0.005) and to 45% after incubation with ALN + OME (*p* ≤ 0.001) (Fig. 2A). ALN induced a rise in OPG after three days (p ≤ 0.001). Incubation with OME and ALN + OME for 24 h reduced the secretion of G-CSF from osteoblasts to 50% (*p* = 0.033) and 30% (*p* = 0.007), respectively (Fig. 2B). ALN induced an increase in G-CSF after three days (*p* = 0.026). Both exposure to OME and ALN + OME reduced the secretion of MCP-1 from osteoblasts to 40% of control after one day of incubation (*p* ≤ 0.001). ALN induced a rise in MCP-1 after three days (*p* = 0.019) persisting to day 7 (Fig. 2C).

### 3.4 Factors affecting inflammation

**Fig. 3:**
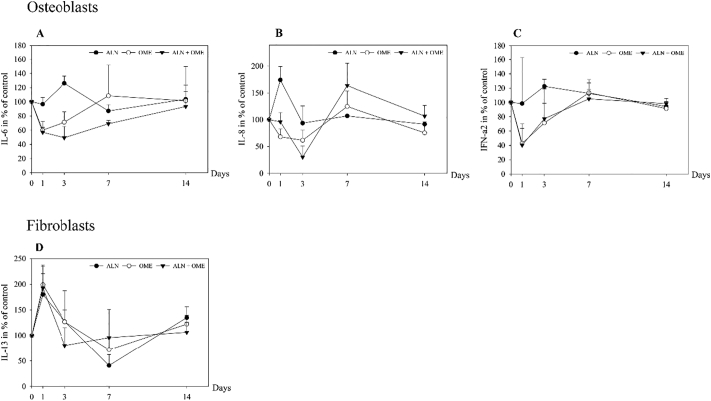
Measured levels of interleukin-6 (IL-6) (A), interleukin-8 (IL-8) (B), interferon-alpha2 (IFN-a2) (C) in media from human osteoblasts and interleukin-13 (IL-13) (D) in cell media from human gingival fibroblasts. The cells were exposed to 5 μM ALN, 1 μM OME and ALN + OME. Data are presented in % relative to untreated control at each time point.

ALN induced a rise in secretion of IL-6 from osteoblasts after three days (*p* = 0.008), whereas a decline to 70% and 50% of control was seen at day three after incubation with OME (*p* = 0.010) and ALN + OME (*p* ≤ 0.001), respectively (Fig. 3A). The secretion of IL-8 from osteoblasts was increased after 24 h of exposure to ALN (*p* = 0.002), whereas OME evoked a decrease after three days of incubation. Exposure to ALN + OME promoted a decline in IL-8 after three days (*p* = 0.006), followed by a rise to 160% of control at day seven (*p* = 0.021) (Fig. 3B). OME reduced the release of IFN-a2 from the osteoblasts to 40% (*p* = 0.020), and ALN + OME to 45% (*p* = 0.017) of control after 24 h of incubation. ALN induced a non-significant rise in IFN-a2 after three days (Fig. 3C). ALN, OME and ALN + OME resulted in a near 2-fold increase in the release of IL-13 from fibroblasts after 24 h (Fig. 3D).

Several of the factors tested were not found to change significantly at any time point (leptin, OC, sclerostin, IL-1ra), or the concentrations were lower than the set levels of detection for the analyses (FGF-23, EGF, eotaxin, IL-10, IL-12p40, IL-15, IL-17, IL-1a, IL-1b, IL-2, IL-3, IL-4, IL-5, IL-7, IP-10, MIP-1a, MIP-1b, TNF-a and TNF-b).

## 4 Discussion

This is the first study to address the effects of concomitant exposure with ALN and OME on cellular functions of human osteoblasts and gingival fibroblasts. We show that the drugs in combination exerted effects that may favor development of ONJ. The combination of ALN and OME induced a marked decline in proliferation of both cell types. The viability of osteoblasts and fibroblasts was unchanged after exposure to ALN, OME or the combination. ALN promoted an initial, transient increase in pro-inflammatory cytokines, whereas OME seemed to act anti-inflammatory. After an initial suppression of IL-8, ALN + OME evoked a rise after seven days. All three drug options stimulated IL-13 release from fibroblasts, followed by a decline. OME both alone and combined with ALN caused an immediate reduction in the secretion of VEGF from the osteoblasts. Moreover, the combination of drugs resulted in a reduced secretion of OPG from osteoblasts and INF-g from fibroblasts.

The majority of cases with MRONJ, including BRONJ, exhibit exposed bone with a duration of more than 8 weeks (Nicolatou-Galitis et al., 2019). Hence, factors that disrupt the process of bone repair may promote ONJ. As a response to physical damage, a cascade of processes is induced to promote bone healing. This is accomplished through complex interactions between inflammatory cells, fibroblasts, osteoblasts, and cells of the monocyte-macrophage-osteoclast lineage. Acute inflammation is the first stage of bone repair, as signified by release of proinflammatory cytokines, including IL-6 (Loi et al., 2016). Thereafter angiogenic and osteogenic pathways are activated. The multifunctional cytokine IL-6 is also involved in promotion of angiogenesis and it seems to be crucial for all stages of fracture healing (Imaculada de Queiroz Rodrigues et al., 2020).

VEGF is released from both osteoblasts and fibroblasts to support the upregulation of angiogenesis (Hu and Olsen, 2016). Furthermore, fibroblasts also increase the secretion of TGF-b, underlining the cells' key role in the formation of new blood vessels (Kellouche et al., 2007). A satisfactory vascularization is a prerequisite for osteogenesis, thus failing to promote this escalation in angiogenesis could result in incomplete healing (Dickson et al., 1994). Notably, VEGF also plays a role in coupling of angiogenesis and osteogenesis, and stimulates bone formation and remodeling (Grosso et al., 2017).

We observed that exposure to ALN induced a rise in several proinflammatory cytokines, including IL-6 and IL-8 from osteoblasts and IL-13 from fibroblasts. This concords with in vivo studies, showing elevation of pro-inflammatory cytokines after treatment with ALN (Morita et al., 2017). IL-13 has been shown to stimulate the formation of the pro-inflammatory cytokine IL-6 (Frost et al., 2001). Markedly increased plasma and salivary levels of IL-6 have been reported in advanced stages of BRONJ (Bagan et al., 2014).

As elaborated on, an initial inflammation also occurs in the bone healing process. Thus, we speculate that the transient rise promoted by ALN may not interfere with bone repair. However, in the case of the systemic and sustained inflammation induced by diseases such as diabetes and rheumatoid arthritis, bone healing may be impaired or delayed, and risk of non-unions is increased (Claes et al., 2012). This concords with the increased risk of BRONJ observed in diabetes patients treated with BPs (Peer and Khamaisi, 2015). Moreover, it is reasonable that BPs may provoke a more pronounced and persistent inflammation in some individuals, hence impeding bone healing. Indeed, inflammation is a hallmark of BRONJ and of ONJ in general (Chang et al., 2018).

Several studies report enhancement of VEGF secretion from osteoblasts and gingival fibroblasts after exposure to BPs (Yuan et al., 2019; Manzano-Moreno et al., 2018). We observed no initial effect on angiogenic factors after exposure of osteoblasts to ALN alone. A rise in VEGF from fibroblasts was seen after 14 days of exposure to ALN and the combined drugs. We interpret this as a secondary response evoked by IL-13, as this cytokine has been shown to stimulate VEGF (Lee et al., 2004). Hence, this may be a mechanism to counteract impairment of angiogenesis, and to favor osteogenesis. Likewise, the increment in IL-6 and IL-8 release from osteoblasts induced by ALN could play a role in maintenance of angiogenesis (Martin et al., 2009; Kayakabe et al., 2012).

Given that these findings translate to in vivo conditions, exposure to ALN alone would affect bone healing modestly. In support of this, a meta-analysis including eight eligible randomized controlled trials with 2508 patients, showed no clinically detectable delay to fracture healing in users of BPs (Xue et al., 2014). It has been postulated that some are predisposed to develop BRONJ in general. Accordingly, a recent study by Lee et al. suggested that patients with impaired function of angiogenesis, osteoclast activity and tissue repair treated with high dose BPs were more prone to BRONJ (Lee et al., 2019). In support of this, some of the candidate genes identified by genome-wide association studies were *TGF-b*, *MMP2*, *PPARG*, *CYP2C8*, *VEGF*, *COL1A1*, *RANK* and *OPG* (Lee et al., 2019).

In contrast to ALN, OME and the combination of drugs promoted transient, inhibitory effects on proinflammatory cytokines (IL-6, IL-8, IFN-a2, MCP-1 and G-CSF) secreted from osteoblasts, whereas an early rise occurred in IL-13 release from fibroblasts. As elaborated on above, IL-13 has been shown to stimulate both IL-6 and VEGF (Frost et al., 2001). Finally, a substantially increased secretion of IL-8 occurred after 7 days of exposure to OME and ALN + OME. IL-8 is one of the major mediators of inflammation (Kany et al., 2019). An initial decline in the angiogenic factors VEGF, IFN-g and IL-6 was induced by OME and the combination with ALN, accompanied by an early rise in IFN-g and a late rise in IL8 and VEGF.

The effects exerted by OME and the combination of drugs could potentially induce impairment of all stages of bone healing. This is supported by a study in mice showing that the PPI pantoprazole affected fracture repair adversely (Histing et al., 2012). Moreover, Subaie et al. demonstrated that omeprazole had a negative effect on bone healing and osseointegration of titanium implants in a rat model (Al Subaie et al., 2016). In both studies, these effects seemed to be mediated through inhibition of bone formation and bone remodeling. It is reasonable that similar mechanisms apply in bone repair and regeneration of the jaw. The interference with factors involved in inflammation, angiogenesis and osteogenesis could under certain circumstances like dental extraction promote ONJ. It should be recalled that ONJ also has been reported in a patient using only PPI (Marconcini et al., 2019), and that the use of PPIs in patients on ALN therapy was linked with surgically treated BRONJ (Eiken et al., 2017).

Factors that affect osteoblast proliferation and differentiation could also interfere with bone healing. Data on the effect of BPs on proliferation of osteoblasts and osteoblast-like cells are diverging. In the present study, ALN induced an initial decline in the proliferation of osteoblasts, followed by an increase after 3 days, and a decrease after 7 days. Koch et al. reported that exposure to zoledronate decreased the proliferation of human osteoblasts (Koch et al., 2010). In contrast, several studies have shown that ALN and other BPs enhance proliferation of osteoblasts and osteoblast-like cells (Krüger et al., 2016; Xiong et al., 2009). This discrepancy may be attributed to differences in the dosage applied and the time points studied. Based on previous in vitro studies, we used a concentration of 5 μM ALN, considered to be in the therapeutic range (Scheper et al., 2009), whereas Koch et al. used 50 μM.

OME evoked a similar pattern as ALN with a transient reduction in proliferation, a rise after 3 days, and a decline after 7 days. Costa-Rodrigues et al. reported that exposure to OME induced an inhibition of both osteoblast proliferation and differentiation (Costa-Rodrigues et al., 2013). Notably, they found a significantly reduced rate of proliferation after exposure to concentrations of 10 μM and higher. In comparison, we used a concentration of 1 μM considered to be at a clinically relevant level (Costa-Rodrigues et al., 2013). ALN and OME in combination induced a decline in osteoblast proliferation at all time points. ALN at a dosage of 2.5 μM has previously been shown to increase proliferation in gingival fibroblasts after one week of exposure (Acil et al., 2018). In the present study, neither ALN, nor OME seemed to affect proliferation of these cells. No previous reports on the effect of OME or other PPIs on fibroblasts were found. The suppression of proliferation of gingival fibroblasts after exposure to both drugs, could result in prolongation of the healing of mucosal and gingival lesions, theoretically stressing the underlying bone (Ravosa et al., 2011).

Osteoclasts are essential for bone homeostasis and fracture healing, and inhibition of osteoclast differentiation has been reported to delay bone healing (Bahney et al., 2019). The OPG/RANKL system plays a major role in osteoclastogenesis. OPG is a decoy receptor that binds RANKL, thereby preventing the binding of RANKL to its receptor RANK and thus reducing the development of osteoclasts (Boyce and Xing, 2007). Several studies, both in vitro and in vivo have reported that BPs promote OPG release from osteoblasts, whereas some observed no effects on the RANKL/OPG system (Kim et al., 2002). However, ibandronate and zoledronate were shown to induce a pronounced enhancement of the RANKL gene expression in human osteoblasts, whereas OPG gene expression was moderately increased (Koch et al., 2012). In concordance with this, we observed that ALN stimulated OPG secretion. On the other hand, OME and the drugs in combination caused an immediate reduction of OPG to less than 50% of control. A similar prompt reduction also occurred in secretion of IFN-g from osteoblasts after exposure to OME and ALN + OME, whereas ALN promoted a rise. The attenuation of both OPG and IFN-g release induced by the combined drugs implies favoring of osteoclastogenesis (Kak et al., 2018). ALN promoted release of factors with opposite effects on osteoclastogenesis, OPG and IFN-g acting inhibitory, and G-CSF and MCP-1 exerting stimulatory effects (Christopher and Link, 2008; Mulholland et al., 2019). The significance of these observations under in vivo conditions is difficult to rule out.

Taken together, the effects evoked by the combined exposure of ALN and OME seemed to be more pronounced than of each drug alone, and may translate to impairment of immune responses, angiogenesis, and osteogenesis in vivo. Under given circumstances, like dental extraction, these changes may affect tissue remodeling and repair adversely, and in some cases lead to BRONJ. The mechanisms revealed may explain the increased risk of ONJ in patients receiving concomitant therapy with BPs and PPIs.

Moreover, recent cohort studies on dental implant outcomes have suggested that PPI intake is associated with an increased risk of implant failure (Altay et al., 2019). Notably, PPI use has also been associated with atypical femoral fracture, another rare adverse effect of BPs (Larsen and Schmal, 2018; Yang et al., 2015). It is reasonable that some of the same mechanisms apply in the pathogenesis of the two conditions. Our findings support the notion that PPIs may further exaggerate the molecular mechanisms that favor development of BRONJ or actually MRONJ. It should be recalled that PPIs also seem to blunt the fracture-reducing effect of ALN (Yang et al., 2015; Abrahamsen et al., 2011). Hence, in patients experiencing gastro-intestinal side effects of oral ALN, treatment with an IV BP should be considered instead of adding a PPI.

We acknowledge that our findings not necessarily translate to an in vivo setting. Most of the effects evoked by the different drug options were short-term, and it may be questioned whether they have pathophysiological consequences in vivo. It is, however, reasonable that the duration of these effects is different and may be more sustained under in vivo conditions. Moreover, the alterations promoted by the drugs are in correspondence with several in vivo studies addressing ONJ (Morita et al., 2017; Xue et al., 2014). Ideally, osteoblasts derived from the mandible should have been applied, as osteoblasts from tibia and the mandible seem to behave a little differently with respect to bone turnover and functionality (Huja and Beck, 2008; Marolt et al., 2012). Furthermore, it would have been beneficial with more than one donor of osteoblasts. We used human primary cells, thus ensuring a cell behavior that reflects the in vivo niche, and that has more preclinical and clinical applicability. The concentration of the drugs was based on what is considered to be the therapeutic level. However, with the relatively high turnover of bone in the jaws and BPs strong affinity for bone matrix, an exact measure of local exposure to the drug is uncertain at best (Khan et al., 1997).

## 5 Conclusion

The combination of ALN and OME seemed to exaggerate the negative effects of each drug alone on human osteoblasts and gingival fibroblasts. The modulation of pro-inflammatory cytokines, impairment of angiogenesis, and anti-proliferative effects may induce conditions in periodontal tissue favoring development of osteonecrosis. Additional studies are needed to evaluate the clinical relevance.

## CRediT authorship contribution statement

**Krüger TB**: main author; done the experiments, evaluated the results and drafted the manuscript, **Herlofson BB**: manuscript revisions and evaluation of results, **Lian AM**: Luminex analysis, **Syversen U**: manuscript revisions and evaluation of results, **Reseland JE**: designed the study, evaluation of results, supervised the experiments, manuscript revisions.

## Declaration of competing interest

The authors declare that they have no known competing financial interests or personal relationships that could have appeared to influence the work reported in this paper.
